# Where to Ride? An Explorative Study to Investigate Potential Risk Factors of Personal Mobility Accidents

**DOI:** 10.3390/ijerph18030965

**Published:** 2021-01-22

**Authors:** Jihun Oh, Jeongseob Kim

**Affiliations:** Department of Urban and Environmental Engineering, Ulsan National Institute of Science and Technology, Ulsan 44919, Korea; wlgnsdl414@unist.ac.kr

**Keywords:** personal mobility, micro-mobility, accident pattern, risk factors, sidewalk

## Abstract

As a mobility of future, the popularity of personal mobility vehicles (PMs) is rapidly increasing worldwide. However, this boom in the use of PMs has resulted in a substantial number of accidents involving not only PM users but also other road users including pedestrians, bicyclists, and motor vehicle drivers. This study aims to explore the potential risk factors for the occurrence of PM-related accidents and the resulting injury severity using the Traffic Accident Analysis System (TAAS) of South Korea between 2017 and 2019. We found that PM–pedestrian accidents tend to occur on roads with wider sidewalks and bike lanes, possibly because the pedestrian–PM conflict increases in this road condition. There is still ongoing debate on whether it is appropriate for PMs to share the sidewalk with pedestrians. Some countries, including Korea, prohibit the use of PMs on sidewalks; however, in reality, this regulation is not well-observed because using PMs on roadways involves higher crash risk with motor vehicles. This study suggests one potential solution to ensure safety of PM users: expansion of bike lane infrastructure having physically separated bike lanes and sidewalks/motorways in addition to the formation and strict enforcement of appropriate safety rules for PM users.

## 1. Introduction

The use of personal mobility vehicles (hereinafter, PMs) is rapidly increasing across the world as a convenient and fairly fast transportation mode for short-distance travel [1]. PMs include a broad range of micro-mobility devices such as electric bike, electric wheel (e.g., hoverboard, Segway), and electric scooter (e-scooter). Leg-kick-type e-scooter (i.e., electric kickboard) along with the shared e-scooter service is fast becoming the most popular type of PM in many countries, including South Korea [2,3]. Being micro-mobility devices supported by electric energy, PMs can reduce greenhouse gas emissions caused by automobiles by providing last-mile solution to improve the convenience of public transit [4,5,6]. Therefore, PMs are expected to play an important role in realizing sustainable urban mobility in the future [7].

While urban transport planners and policymakers have welcomed PMs [5,8], there is an ongoing debate on how to create a safe transportation environment for the use of PMs. With the increase in the use of PMs, a rapid growth in PM-related accidents has been witnessed across countries. In the U.S., the number of e-scooter-related injuries was 4583 in 2014, which tripled to 14,641 in 2018 [9]. According to the Korean Consumer Agency, safety accidents involving PMs have increased significantly from 3 in 2013 to 174 in 2016, and the number of traffic accidents officially reported by the police has also doubled to 225 from 117 in 2017 [10]. Besides the growing number of PM-related accidents, PMs are viewed as an unwelcome intruder affecting other road users, especially pedestrians. Pedestrians often feel it dangerous to share sidewalk spaces with PMs [11,12,13]. However, there is no agreement on safety guidelines as to whether it is appropriate for PMs to use sidewalks.

As the safety issues concerning PMs intensify, countries worldwide are taking preventive measures against PM-related traffic accidents. The regulations on PMs currently being implemented are focused more on the users, mainly including limits on driving speed, where to ride, age eligibility for PMs use, and other safety codes like wearing a helmet [14]. European cities, in particular, are making efforts to limit the use of PMs in pedestrian areas: France banned electric scooters from pavements in September 2019; instead, the users will have to use bikeway or (if non-applicable) roadway. In the U.K. and Germany, riding PMs on sidewalks is now illegal. Sweden has introduced a 20 km/h speed limit for PMs in bike lanes in cities [14].

While cities around the world are trying to develop appropriate preventive measures against PM-related accidents, little is known about the characteristics of such accidents as well as the factors contributing to these accidents. Some researchers have reported PM usage and injury patterns. Regarding the usage pattern of PMs, e-scooters are commonly used for leisure and recreational purposes and are less likely to be used for commuting [15,16,17]. In contrast, Nishad, Padhya, and Gandhi [7] report that PMs are used particularly for commuting trips. This difference in findings might be attributed to the fact that transport infrastructure and institutional environment vary from city to city. On the other hand, researchers in the medical field mainly focused on the injury patterns resulting from PM-related accidents. The findings of these studies, conducted in various countries, are quite consistent: first, among those injured, riders were mainly young men aged less than 30 years [18,19] and non-riders were represented by elderly women [20,21], second, head injuries were the most common [9,22], and third, most of the riders did not wear a helmet [23], whereas some rode under the influence of alcohol [24]. Although these studies provide knowledge regarding the occurrence and severity of PM-related accidents, they do not systematically address the variety of physical, locational, and situational factors behind PM-related accidents [25].

Investigation on where, when, and how the accidents take place needs to be conducted in order to implement the countermeasures to minimize the risks posed by PMs, which might lift the intention to use this transport mode and reduce burden on the urban transport system [26]. The bicycle is probably the most similar to PMs in that it is another prominent type of micro-mobility device and has quite similar driving capabilities [14]. The knowledge about their usage patterns and risks has been accumulated through a large body of literature, followed by actual policy implementations for integrating bicycles into urban road spaces. Reynolds et al. [27] reviewed existing studies and identified risk factors determining injuries and crashes among bicyclists, especially in terms of the built environment: first, multi-lane roundabouts can significantly increase risk to bicyclists unless a separated cycle track is included in the design, as further examined in Reference [28], second, sidewalks and multi-use trails (i.e., sidewalks for both pedestrians and bicyclists) pose the highest risk, see Reference [29], and third, the presence of bicycle facilities (e.g., on-road bike routes, on-road marked bike lanes, and off-road bike paths) was associated with the lowest risk. Billot-Grasset et al. [30] emphasize that improved infrastructure design and maintenance is needed to avoid falls and collisions with obstacles. Accordingly, each city has developed safety policies and designs for bicycles based on this knowledge, and as Rietveld and Daniel [31] revealed, each city’s policy-related contexts (e.g., quality/capacity of bicycle-dedicated infrastructure and safety regulation) are highly interconnected with the number of bicyclists and their safety.

In contrast, only a few studies tried to identify the potential risk factors behind PM-related accidents with regard to the situational elements. Kim et al. [32] found that the majority of PM-related accidents occurred on the sidewalk, street, and the alley. The riders fell off on both sidewalks and arterial roads/streets, which could be caused by multiple factors such as riding on uneven road pavements, avoiding fixed objects (e.g., trees), conflicts with pedestrians and vehicles, etc. [1]. With the low visibility of the riding environment, a higher proportion of reported fatal crashes occurred at night-time [1,32]. Stormann, Klug et al. [19] found that, compared to weekdays, the weekend is related to a higher probability of PM-related crashes. To sum up, while PMs are now one of the familiar road transports in urban life, the risks associated with PMs remain unknown.

Therefore, this study aims to explore the potential risk factors for the occurrence of PM-related accidents and the resulting injury severity. Specifically, three research questions are addressed: (1) What are the locational and environmental characteristics of PM-related accidents and their injury severity? (2) What are the similarities and differences in the risk factors between PM- and bicycle-related accidents? (3) Are there any differences in potential risk factors between PM–motor vehicle accidents and PM–pedestrian crashes?

To answer these research questions, this study analyzes PM-related accidents in South Korea by comparing them with bicycle-related accidents. The differences between this study and the previous ones are three-fold: first, we comprehensively consider not only the accident characteristics but also a variety of environmental and locational factors associated with accident occurrence and the resulting severity. Second, we investigate the PM-related accident patterns mainly in comparison with bicycle accidents. According to Verstappen et al. [33], there are no significant differences in the mechanism and severity of injury between PM- and bicycle-related accidents except for a higher rate of thoracic and soft-tissue trauma in the PM riders. By comparing the accident patterns between PMs and bicycles, implications regarding safety guidelines specialized for PM users could be suggested. Third, by exploring different potential risk factors for each type of PM-related accident, this study adds to the understanding of various patterns of PM-related accidents.

## 2. Materials and Methods

### 2.1. Data and Variables

We collected the accident data officially registered by the National Police Agency (NPA) between the years 2017 and 2019 from the Traffic Accident Analysis System (TAAS) of South Korea. Considering the growth in PM-related accidents, the NPA added a PM-related accident category in 2017 and made available the related accident data since 2017 through TAAS. The definition of PMs in TAAS includes various types of micro-mobility devices; however, e-scooters are the most popular because of the shared e-scooter mobility service. In the TAAS, mobility scooters (i.e., electric wheelchair) are not considered PMs because they fall into a pedestrian category as an essential assistance device for the elderly or disabled in Korea. Since electric bicycles are considered as bicycles in the TAAS, accidents related to electric bicycles have been included in bicycle accidents. From the accident data, we filtered the PM- and bicycle-related accidents in which the offender/victim was a PM and bicycle rider respectively, and excluded cases with missing information. Through the data mining process, 1488 PM-related and 36,586 bicycle-related accidents within the study period were selected. It is noteworthy that the accidents database in TAAS includes all traffic accidents handled by policy officers or insurance companies regardless of being reported to police stations. The accident information contains the XY coordinates of accident location. Based on the location, we merged the TAAS accident data with other various spatial data, such as road and land use characteristics. A detailed description about the variables is presented in Table 1. The database created thus includes not only the accident characteristics but also the situational characteristics, such as environmental and locational factors, which can contribute to the occurrence and severity of the accident. In particular, detailed road attributes were included as the main variable for locational characteristics. To investigate the role of road infrastructure in accident risk, we categorized roads into six types: (1) road without sidewalk, (2) road with sidewalk only, (3) road with sidewalk and bikeway, (4) pedestrian-only road, (5) bicycle lane, and (6) off-road. Further, we included the width of road and pavement status.

### 2.2. Methods of Analysis

The overall characteristics of PM-related accidents are presented using descriptive statistics. To measure the spatial relationship between the location of accidents and population distribution, we compared the spatial characteristics by mapping the variables and calculating the Pearson correlation coefficients between each distribution. To present the patterns of PM-related accidents compared to bicycle-related accidents, this study applies the unequal variance two-tailed t-test between the frequency of PM-related and bicycle-related accidents for a given category. In situations where exposure is not controlled, comparison of each risk factor within PM-related accidents could provide false information about accident risk. Therefore, we focused on the comparison of risk factors between PM-related and bicycle-related accidents. In other words, this study does not develop an accident occurrence model because there are no available datasets for the volume of PM users or bicycle users. Thus, instead of developing the accident occurrence model, we focus on the distinctive variables differentiating PM accidents from bicycle accidents and conduct detailed descriptive analysis to identify the potential risk factors related to accident occurrence with an emphasis on road characteristics. We further discuss the differences in potential risk factors by comparing PM–motor vehicle accidents and PM–pedestrian crashes. Lastly, we build the injury severity model of PM-related accidents using an ordinal logit regression model to examine the determining factors of victim’s injury severity for two accident types (PM with motor vehicle/PM with pedestrian).

## 3. Results

### 3.1. Trends and Characteristics of PM Accidents

The trends of PM-related accidents between 2017 and 2019 are summarized in Table 2 compared with the case of motor vehicles and bicycles. During the period, the number of accidents involving with motor vehicle increased by 7.1% and bicycle-related accidents reduced by 6.1%. Compared to these two types, the PM accidents have increased by 282% from 216 to 823. Although it is estimated by sales number of PM rather than actual number of its users due to the lack of data availability, considering the number of potential users, the accident incidence ratio for PM accidents has been rapidly increased compared to motor vehicle or bicycle accidents. It indicates that both the use of PM and related accidents are increasing rapidly.

The reason why we focused on Seoul to analyze the spatial patterns of PM-related accidents is that being the capital city of South Korea, many companies are providing shared personal mobility services in Seoul, and subsequently, Seoul accounts for the largest number of PM-related accidents in Korea. Every one in four PM-related accidents occur in Seoul. Considering the small number of PM-related accidents overall, spatial analysis at the national level would not provide meaningful results. As noted earlier, we compared the spatial distribution of accident locations with population and floating population using maps and Pearson correlation coefficients.

**Figure 1:**
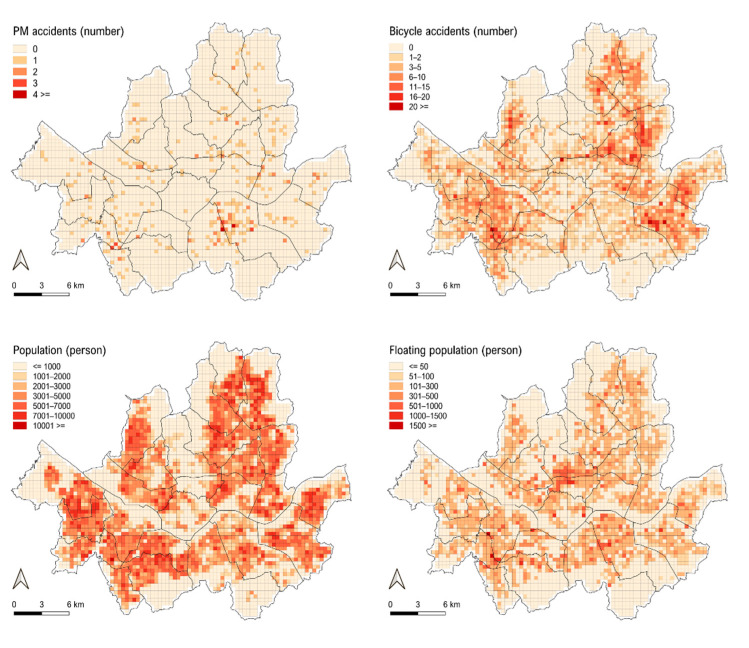
Spatial accident pattern.

As shown in Figure 1, compared to bicycle accidents, PM accidents are concentrated in specific areas, possibly because the location of PM accidents is related to the shared PM service zones in Seoul. According to the correlation coefficients in Table 3, PM accidents are more likely to be positively related with floating population, while bicycle accidents are more likely to be connected with population. The coefficients between the location of PM accidents and floating population are about 1.5 times higher than that between PM accidents and population. Bicycle accidents occurred frequently near residential zones, suggesting that people often use bicycles near their residence. In contrast, PM accidents mainly occurred in areas with large floating population. The spatial pattern of PM accidents in Seoul might change when the use of PMs becomes much more common across the city in the future, but the current pattern itself is meaningful in that it represents the spatial patterns of PM accidents in the early stages of their introduction.

The overall accident characteristics of PM-related accidents are summarized in Table 4. More than three-fourths of PM accidents are related to motor vehicles. PM accidents with pedestrians are about two times higher than those with bicycles, implying that there is more conflict between PM users and pedestrians on the roadways. The higher conflict rate with pedestrians suggests that PMs are riskier than bicycles for pedestrians even though they have quite similar driving capabilities. Similarly, the share of self-accidents in PM accidents is more than three times that in bicycle accidents, indicating inexperienced driving could be more frequent among PM users compared to bicycle users. Relatively younger people aged between 16 and 49 are more likely to experience PM accidents compared to bicycle users. It makes sense because PMs have become popular mainly among young people as next-generation transport, whereas the bicycle is a classic transportation mode used by all age groups. Only the group aged less than 16 showed a lower share of PM accidents. This is because the use of PMs is restricted by the law in Korea if a person has no driving license for any kind of automobile. The level of injury severity is quite similar between PM and bicycle accidents.

### 3.2. Factors Affecting PM Accidents

The results of the two-tailed t-test for comparing PM and bicycle accidents are presented in Table 5. As PM accidents with motor vehicles consist of 76% of all PM accidents, the accident characteristics of this type are similar to the overall accident characteristics of PM. Further, compared to bicycle accidents with motor vehicles, injuries leading to death are relatively lower in PM accidents with motor vehicles. PM accidents with motor vehicles occur more frequently at night-time than bicycle accidents, possibly because of a lack of safety regulations on the use of PMs at night-time. The share of accidents on roads with both sidewalk and bikeway is relatively lower in PMs than bicycles. Also, PMs have higher share of off-road accidents than bicycles. Compared to bicycles, PM accidents with motor vehicles are more likely to occur on wider roads, which possibly relates to the higher PM user volume in commercial zones.

The accident characteristics of PMs with pedestrians are different from those with motor vehicles. PM accidents with pedestrians tend to be caused more by women than men. Women’s share in such accidents is higher than that in PM–motor vehicle accidents as well as in bicycle–pedestrian accidents. The differences by age groups between PM–pedestrian and bicycle–pedestrian accidents were found, but these patterns may be related to the user volume, not controlled in the t-test. PM–pedestrian accidents are more likely to have lower injury severity than bicycle–pedestrian accidents. The share of minor injury in PM–pedestrian accidents is 12% higher than that in bicycle–pedestrian accidents. Another interesting finding from the t-test is that PM–pedestrian accidents tend to occur on roads with sidewalk (32%), whereas only 17% of bicycle–pedestrian accidents occur on this type of road. This indicates that the conflict between PM users and pedestrians is severe. In Korea, the use of sidewalk by PMs is prohibited by the law, but, in practice, this rule is not followed well, and its enforcement is weak. In the absence of bicycle lanes, PM users tend to use sidewalks instead of motorways, leading to more frequent accidents with pedestrians.

In both PM–motor vehicle and PM–pedestrian accidents, the road type is an important factor that results in the unique characteristics of PM accidents. Therefore, additional analysis on how the road type and road width are related to the incidence of PM accidents was conducted. Specifically, we calculated the sidewalk width for each road to examine whether the existence of sidewalk and bikeway affects the frequency of PM and bicycle accident occurrence. The width of sidewalk was estimated based on the road width and the number of road lanes considering the design guideline of the bicycle lane installment manual [34]. We categorized the sidewalks into four types on the basis of width: (1) less than 2 m, (2) 2–3.49 m, (3) 3.5–4.99 m, and (4) 5 m or wider. According to Seoul City’s bicycle lane installment manual [34], the width of a one-way bicycle lane is often 1.2–1.5 m. Thus, the sidewalks with width 3.5 m or more could have a separate bicycle lane.

**Figure 2:**
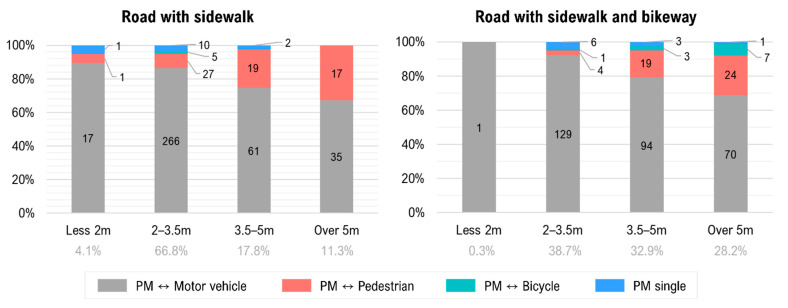
Accident frequency ratio of each accident type by sidewalk width.

Though PM–motor vehicle accidents dominate in all cases, the share of PM–pedestrian accidents increases with the increase in the width of the sidewalk, as shown in Figure 2. As noted earlier, in Korea, the use of PMs on both sidewalk and bicycle lane is restricted by law, and PM users have to use the motorways. However, PM users tend to use the sidewalk when the sidewalk is wider and there is room to share the sidewalk space with pedestrians, thus increasing pedestrian accidents. A similar pattern is observed for roads with both sidewalk and bikeway. Interestingly, the share of PM–pedestrian accidents is relatively lower in roads with sidewalk and bikeways, while the PM–bicycle accidents increase. This is because when a bike lane is available, PM users choose to use the bicycle lane instead of the sidewalk, consequently reducing the potential conflicts between PM and pedestrians.

In addition, the pattern of PM–pedestrian accidents vary depending on each road type (Table 6). Overall, about 23% and 21% of the PM–pedestrian accidents occurred while the pedestrians were using the sidewalk and crossing the road, respectively. About 21% of PM–pedestrian accidents occurred on the roadway walking or in crossing the road. In the roadway without sidewalk, the PM–pedestrian crashes frequently occurred when the pedestrians were walking on roadway (23.8%). On the other hand, the share of PM–pedestrian crashes during walking on sidewalk was highest for roads with both sidewalk and bicycle lanes. These results imply that the conflicts between PMs and pedestrians on sidewalk are one of the main reasons behind PM–pedestrian crashes.

### 3.3. Injury Severity Modeling

We fitted two ordinal logit regression models where the dependent variable is injury severity level (1 = No injury, 2 = Wound, 3 = Minor injury, 4 = Severe injury, 5 = Death) for PM–motor vehicle and PM–pedestrian crashes by using the polr function in R. The subject of injury severity for each accident type has been defined as PM rider in the PM–motor vehicle accident and pedestrian in the PM–pedestrian accident. We reported the estimated odds ratios of the regression model. If an odds ratio of a variable is larger than 1, the variable increases the injury severity, and vice versa.

Regarding the injury severity of PM–motor vehicle accidents, Table 7 shows that the severity increases when the PM user is the victim of the accident rather than an offender. Specifically, when the PM user is the victim of the accident, the injury severity is 3.597 times higher than that of the offender. The age and gender of PM user, and time and environmental characteristics, do not have statistically significant effects on the injury severity, except for accidents on rainy/snowy days. Not surprisingly, the bad weather condition increased the severity of PM–motor vehicle accidents. Interestingly, the injury severity of PM–motor vehicle accidents is higher when the crash occurs in rural areas or on unpaved roadways or in green/rural land use zones.

These results are possibly because the drivers of motor vehicles are less likely to use cautious driving and are not expecting the use of PMs on the roadway in rural environments. PM crashes in rural areas or rural/green zones consist of about 7.7% and 17.3% of all PM crashes, respectively. Considering the fact that PMs tend to be used for recreational purposes and the traffic volume of PM riders is relatively lower in these areas, motor vehicle drivers may not expect to meet PM riders on the streets, resulting in less cautious driving. Road characteristics are important factors to determine the frequency of PM–motor vehicle accidents, but they do not have statistically significant effects on the severity of PM–motor vehicle accidents.

The factors affecting the injury severity of PM–pedestrian accidents are different from those of PM–motor vehicle accidents. In PM–pedestrian crashes, injury severity is higher when the pedestrian is elderly, or when the accident occurs at night-time. Further, in contrast to PM–motor vehicle accidents, the injury severity of PM–pedestrian crashes is higher in urban areas. Regarding the road characteristics, the injury severity of PM–pedestrian crashes increases when the accidents occur on roads with both sidewalks and bikeways. In Korea, the bikeways and sidewalks are often color-coded to indicate their separate uses rather than being separated physically. In such a condition, an accident can occur if PM riders using the bikeways and pedestrians using the sidewalk are not mindful of the color-coded separation of spaces. As for the road types, accidents occurring on narrow roads tend to result in severe injury. There might not be enough space on narrow roads for PM users and pedestrians to try to avoid accidents, which can lead to more serious accidents.

## 4. Discussion

There are several distinctive patterns that are observed in PM accidents in comparison with bicycle accidents. First of all, and most importantly, PM–pedestrian accidents occur more frequently (14.2%) than bicycle–pedestrian accidents (7.6%), indicating higher conflict with pedestrians in PM-related accidents. Further, the share of PM–pedestrian accidents increases as the width of sidewalk, including the width of bike lane, increases. In addition, the injury severity of PM–pedestrian crashes is higher in this road type. These findings might be attributed to the lack of a systematic legal system for the use of PMs on the streets. Moreover, following the rules, bicycle users only use bicycle lanes, whereas PM users tend to use both bicycle lanes and sidewalks. Under the law, PM users are allowed to use only roadways; however, law enforcement is weak. Further, when PM users use the roadway, the risk of accident with motor vehicles increases; therefore, they tend to choose the relatively safe sidewalk.

In consideration of this situation, Korea recently revised the law to allow PM users to use bicycle lanes; that is, when bicycle lanes are available, PM users can use the lanes, but they should not use the sidewalk, and if there are no bicycle lanes, PM users must use the roadways. The policy office plans to enforce the PM travel rules with the help of increased penalties for violation. Considering that PM users prefer to use the sidewalk, systematic monitoring is required for the safety-enhancing effects of changed regulations to become visible.

Another distinctive pattern of PM-related accidents is a relatively higher frequency of off-road accidents (13.3% of PM accidents vs. 9.5% of bicycle accidents). In fact, according to a survey on PM usage pattern conducted by Korea Road Traffic Authority Traffic Science Institute (KRTATSI), 35.3% of PM users responded that they mostly use off-road such as parks or trails [12]. Of all PM–pedestrian or PM–bicycle accidents, 40.1% were off-road accidents, whereas 14.1% occurred on-road. Table 8 lists in detail the specific locations where off-road accidents took place. Since there is no available information about off-roads, we manually searched each accident location and assigned it to the corresponding off-road category. Interestingly, PM accidents frequently occurred in parking lots or within apartment complexes. This indicates that proper safety guidelines for PM users should be prepared not only for roadways but also for different types of off-roads.

As PMs are an emerging mobility mode, safety guidelines or rules for riding PMs are not yet in place in many countries. Moreover, there are ongoing debates on whether it is appropriate for PMs to share the sidewalk with pedestrians. Some countries, including Korea, prohibit the use of PMs on sidewalks; however, this regulation is not well-observed in reality as using PMs on roadways involves higher crash risk with motor vehicles. Consequently, PMs are considered unwelcome intruders by other road users.

## 5. Conclusions

This study explored the potential risk factors of PM-related accidents by comparing them with bicycle-related accidents and suggests that the conflicts between PMs and pedestrians on sidewalks should be appropriately addressed by enforcing safety regulations and rules for using PMs. In Korea, riding PMs on sidewalks is illegal. However, considering the fact that the majority of PM accidents occur with motor vehicles (76.1%), the adequacy of this rule should be examined to achieve safer road environment for every transportation mode. Sharing sidewalks between PM users and pedestrians may have the advantage of reducing PM–motor vehicle accidents, but there is a trade-off in that it increases PM–pedestrian crashes. Therefore, if a city government encourages the use of PMs as a sustainable urban mobility mode, regulations on PM use and expansion of PM infrastructure are required to reduce PM–motor vehicle and PM–pedestrian crashes. One possible solution is to prohibit the use of sidewalks by PMs and expand bicycle road infrastructure to allow PM users to share the space with bicycle users. To this end, bicycle lanes need to be designed in such a way that they are physically separated from both the sidewalk and the motorway. Of course, a strict law enforcement of safety rules, such as wearing a helmet and installing lighting devices for night-time use, and speed limit policy can further contribute to reducing PM-related accidents.

As the popularity of PMs continues to rise, the number of related accidents is also increasing. Although many countries around the world have introduced various guidelines and regulations for PM use, there is still a lack of empirical analysis to examine their appropriateness. The findings of this study could be a basis to expand the understanding of PM-related accidents. As data on PM-related accidents and the exposure control for PM users become available, analysis of “actual” risk factors of accident rather than “potential” risk factors should be conducted in the future studies. Further, the outcomes of different regulations on PM use could be monitored and evaluated to ensure safe use of PMs. As the maximum speed varies depending on the type of PM, different accident characteristics by the types of PM could be explored.

## Figures and Tables

**Table 1:** Description of variables used in the analysis.

Variables		Description	Sources
Accident characteristics	Accident type	(1) Accident with motor vehicle, (2) Accident with pedestrian, (3) Accident with bicycle, (4) Self-accident	Traffic Accident Analysis System
	Offender vs. Victim	Whether the PM (Bike) user was offender or victim	
	Gender of PM (Bike) user	(1) Male, (2) Female	
	Age of PM (Bike) user	(1) Less than 16, (2) 16–64, (3) 65 or more	
	Injury severity of PM (Bike) user	(1) No injury, (2) Wound, (3) Minor injury, (4) Severe injury, (5) Death	
	Gender of victim *	(1) Male, (2) Female	
	Age of victim *	(1) Less than 16, (2) 16–64, (3) 65 or more	
	Injury severity of Victim *	(1) No injury, (2) Wound, (3) Minor injury, (4) Severe injury, (5) Death	
Environmental factors	Day of week	(1) Weekday, (2) Weekend/Holiday	
	Time	(1) Daytime (After 6 a.m.–Before 6 p.m.), (2) Night-time (After 6 p.m.–Before 6 a.m.)	
	Season	(1) Spring, (2) Summer, (3) Autumn, (4) Winter	
	Weather	(1) Clear, (2) Cloudy, (3) Rain/Snow	
Locational factors	Region	(1) Urban area, (2) Rural area	Digital map (National Geographic Information Institute)
	Road type	(1) Road without sidewalk, (2) Road with sidewalk only, (3) Road with sidewalk and bikeway, (4) Pedestrian-only road **, (5) Bicycle lane *, (6) Off-road ***	
	Road width	(1) Less than 8 m, (2) 8–12 m, (3) 12–25 m, (4) 25 m or wider, (5) Not Applicable (Off-road)	
	Road intersection ****	(1) Non-intersection, (2) Intersection, (3) Not Applicable (Off-road)	
	Road pavement	(1) Paved, (2) Unpaved, (3) Not Applicable (Off-road)	
	Land slope	(1) Flat (less than 5°), (2) Mild slope (5–15°), (3) Steep slope (15° or higher)	Topographic map (National Institute of Agricultural Sciences)
	Land use	(1) Residential zone, (2) Commercial zone, (3) Rural/Green zone	Land use map (National Spatial Data Portal)

Note: * Victim refers to the personal mobility vehicle (PM) (Bike) user in the cases of accidents with motor vehicle and pedestrian in the cases of accidents with pedestrian. ** No information is available about the width of pedestrian-only roads and bicycle lanes; therefore, we assume their width to be less than 8 m. *** Off-road refers to an area that is not a road, such as parks, parking lots, plaza, university campus, as well as the inside of an apartment complex. **** At intersection, the road characteristics follow the nearest road among the intersected roads.

**Table 2:** Accident trend.

**Accident** **Category**	**(1) Number of Accidents (Unit: Accidents)**			
	**2017**	**2018**	**2019**	**2017–2019**
Motor vehicle	190,095	193,072	203,644	7.1%
Bicycle	13,118	11,148	12,320	−6.1%
Personal mobility (PM)	216	449	823	282.9%
**Accident** **Category**	**(2) Number of Potential Users (Unit: 1000 Users)**			
	**2017**	**2018**	**2019**	**2017–2019**
Motor vehicle	22,530	23,200	23,680	5.1%
Bicycle	13,353	13,353	13,353	0.0%
Personal mobility (PM)	98	167	196	101.2%
**Accident** **Category**	**(3) Incidence Ratio** **(Unit: Accidents per 1000 Users for Motor Vehicle and Bicycle;** **Accidents per 1000 Vehicles for PMs)**			
	**2017**	**2018**	**2019**	**2017–2019**
Motor vehicle	8.4	8.3	8.6	1.9%
Bicycle	1.0	0.8	0.9	−6.1%
Personal mobility (PM)	2.2	2.7	4.2	89.3%

Source: (1) Number of registered motor vehicles (Ministry of Land, Infrastructure and Transport, 2017–2019). (2) Number of bike riders who ride at least once a month (The Korea Transport Institute, November 2016). (3) Accumulated number of PMs sold (Korea Smart E-Mobility Association, 2017–2019).

**Table 3:** Pearson correlation coefficients between accidents and population.

Variables	PM Accident	Bicycle Accident	Population	Floating Population
PM accident	1	0.156	0.131	0.196
Bicycle accident		1	0.358	0.269
Population			1	0.247
Floating population				1

Source: (1) Population: Population and Housing Census (2015). (2) Floating population: Seoul Statistics (2012–2015).

**Table 4:** Characteristics of PM- and bicycle-related accidents.

Variables			PM Accident	Bicycle Accident
Accident characteristics	Accident type	Accident with motor vehicle	76.1%	86.0%
		Accident with pedestrian	14.2%	7.6%
		Accident with bicycle	4.2%	4.7%
		Self-accident	5.6%	1.7%
	Offender vs. Victim	Offender	49.2%	43.3%
		Victim	50.8%	56.7%
	Gender of PM (Bike) user	Male	80.1%	79.6%
		Female	19.9%	20.4%
	Age of PM (Bike) user	Less than 16	3.3%	17.1%
		16–24	19.2%	10.2%
		25–34	25.5%	6.7%
		35–49	28.6%	13.9%
		50–64	15.7%	23.8%
		65 or more	7.8%	28.3%
	Injury severity	No injury	1.3%	0.4%
		Wound	10.5%	12.3%
		Minor injury	54.8%	50.5%
		Severe injury	32.4%	35.0%
		Death	1.1%	1.8%

**Table 5:** Results of two-tailed *t*-test for comparing PM and bicycle accidents.

Variables			Accident with Motor Vehicle			Accident with Pedestrian		
			PM (a)	Bicycle (b)	Differences (a–b)	PM (c)	Bicycle (d)	Differences (c–d)
Accident characteristics	Offender vs. Victim	Offender	34.5%	34.1%	0.4%	100.0%	100.0%	0.0%
		Victim	65.5%	65.9%	−0.4%	0.0%	0.0%	0.0%
	Gender of PM (Bike) user	Male	80.3%	78.7%	1.6%	73.5%	87.3%	−13.9% ***
		Female	19.7%	21.3%	−1.6%	26.5%	12.7%	13.9% ***
	Age of PM (Bike) user	Less than 16	2.8%	17.3%	−14.5% ***	5.7%	20.0%	−14.3% ***
		16–24	17.8%	9.1%	8.6% ***	28.9%	19.3%	9.6% *
		25–34	25.4%	6.5%	19.0% ***	27.5%	6.7%	20.8% ***
		35–49	28.9%	13.1%	15.8% ***	20.9%	18.0%	2.9%
		50–64	16.7%	24.0%	−7.3% ***	10.9%	21.3%	−10.4% **
		65 or more	8.4%	30.0%	−21.6% ***	6.2%	14.7%	−8.5% *
	Injury severity of PM (Bike) user	No injury	4.7%	1.8%	2.9% ***	2.4%	4.7%	−2.3%
		Wound	9.9%	11.7%	−1.8%	11.4%	10.7%	0.7%
		Minor injury	54.1%	51.1%	2.9%	56.9%	44.0%	12.9% *
		Severe injury	30.6%	33.7%	−3.1%	28.9%	40.7%	−11.8% *
		Death	0.8%	1.8%	−1.0% *	0.5%	0.0%	0.5%
Environmental factors	Day of week	Weekday	74.6%	74.6%	0.1%	71.6%	69.3%	2.2%
		Weekend/Holiday	25.4%	25.4%	−0.1%	28.4%	30.7%	−2.2%
	Time	Daytime	67.6%	84.4%	−16.8% ***	77.7%	78.7%	−0.9%
		Night-time	32.4%	15.6%	16.8% ***	22.3%	21.3%	0.9%
	Season	Spring	17.8%	28.0%	−10.3% ***	19.4%	23.3%	−3.9%
		Summer	29.0%	30.4%	−1.4%	35.1%	30.0%	5.1%
		Autumn	36.8%	28.3%	8.6% ***	34.1%	36.7%	−2.5%
		Winter	16.4%	13.3%	3.1% *	11.4%	10.0%	1.4%
	Weather	Clear	95.1%	94.7%	0.3%	95.7%	98.0%	−2.3%
		Cloudy	2.0%	2.3%	−0.3%	1.9%	0.7%	1.2%
		Rain/Snow	2.9%	3.0%	−0.1%	2.4%	1.3%	1.0%
Locational factors	Region	Urban area	92.0%	83.5%	8.5% ***	96.2%	92.0%	4.2%
		Rural area	8.0%	16.5%	−8.5% ***	3.8%	8.0%	−4.2%
	Road type	Road without sidewalk	31.5%	27.8%	3.7% *	19.9%	22.0%	−2.1%
		Road with sidewalk	32.3%	35.7%	−3.3%	31.8%	17.3%	14.4% **
		Road with sidewalkand bikeway	25.1%	30.4%	−5.3% **	19.9%	20.7%	−0.8%
		Pedestrian-only road	0.0%	0.0%	0.0%	10.9%	10.0%	0.9%
		Bicycle lane	0.0%	0.0%	0.0%	0.0%	0.0%	0.0%
		Off-road	11.0%	6.2%	4.9% ***	17.5%	30.0%	−12.5% **
	Road width *	Less than 8 m	16.8%	11.2%	5.6% ***	23.6%	28.6%	−5.0%
		8–11.9 m	20.1%	22.2%	−2.1%	16.1%	17.1%	−1.1%
		12–24.9 m	29.0%	36.2%	−7.2% ***	23.0%	27.6%	−4.6%
		25 m or wider	34.2%	30.4%	3.7% *	37.4%	26.7%	10.7%
	Road intersection ^1^	Non-intersection	38.6%	39.5%	−0.9%	77.6%	77.1%	0.4%
		Intersection	61.4%	60.5%	0.9%	22.4%	22.9%	−0.4%
	Road pavement ^1^	Paved	99.9%	99.9%	0.0%	82.5%	70.0%	12.5% **
		Unpaved	0.1%	0.1%	0.0%	0.0%	0.0%	0.0%
	Land slope	Flat	89.9%	89.7%	0.2%	88.6%	83.3%	5.3%
		Mild slope	9.3%	8.7%	0.6%	11.4%	14.0%	−2.6%
		Steep slope	0.8%	1.6%	−0.8% *	0.0%	2.7%	−2.7% *
	Land use zone	Residential zone	64.9%	62.6%	2.3%	61.6%	51.3%	10.3%
		Commercial zone	19.9%	16.0%	3.8%	23.7%	15.3%	8.4% *
		Rural/Green zone	15.2%	21.3%	−6.1%	14.7%	33.3%	−18.6% ***
N			1132	17,966		211	1583	

Note: *, **, *** refer to the statistical significance at the 5%, 1%, and 0.1% level, respectively. ^1^ For road characteristics, we compare all the factors except the “off-road” factor.

**Table 6:** Frequency of PM–pedestrian accidents by crash situations and road types.

Crash Situations	Road Type				
	Road without Sidewalk	Road with Sidewalk	Road with Sidewalk and Bikeway	Others	Total
Walking on sidewalk	1 (2.4%)	16 (23.9%)	13 (31.0%)	18 (30.0%)	48 (22.7%)
Walking on roadway	10 (23.8%)	1 (1.5%)	2 (4.8%)	3 (5.0%)	16 (7.6%)
Walking on roadside	5 (11.9%)	0 (0.0%)	0 (0.0%)	1 (1.7%)	6 (2.8%)
Crossing	2 (4.8%)	25 (37.3%)	12 (28.6%)	6 (10.0%)	45 (21.3%)
Unknown	24 (57.1%)	25 (37.3%)	15 (35.7%)	32 (53.3%)	96 (45.5%)
Total	42 (100.0%)	67 (100.0%)	42 (100.0%)	60 (100.0%)	211 (100.0%)

**Table 7:** Estimated results of injury severity model of PM accidents.

Variables		Accident with Motor Vehicle (Y = PM’s Injury Severity)		Accident with Pedestrian (Y = Pedestrian’s Injury Severity)	
		Odds Ratio	*z* Value	Odds Ratio	*z* Value
Offender vs. Victim (1 = Victim, 0 = Offender)		3.597	9.374 ***		
Gender of victim (1 = Female, 0 = Male)		0.948	−0.354	1.530	1.346
Age of victim	16–64 (ref.)				
	Less than 16	0.548	−1.666	0.650	−0.878
	65 or more	1.368	1.391	2.748	3.033 **
Day of week (1 = Weekend/Holiday, 0 = Weekday)		0.998	−0.015	0.948	−0.165
Time (1 = Night-time, 0 = Daytime)		0.926	−0.589	2.409	2.356 *
Season	Spring (ref.)				
	Summer	0.813	−1.181	1.361	0.728
	Autumn	0.997	−0.021	0.845	−0.404
	Winter	0.733	−1.570	1.549	0.788
Weather	Clear (ref.)				
	Cloudy	1.479	0.912	0.342	−0.836
	Rain/Snow	1.854	1.862 *	0.488	−0.784
Region (1 = Rural area, 0 = Urban area)		1.586	1.984 *	0.220	−2.008 *
Road type	Road without sidewalk (ref.)				
	Road with sidewalk	0.902	−0.346	4.277	1.871
	Road with sidewalk and bikeway	0.954	−0.143	5.520	2.401 *
	Pedestrian-only road			0.315	−1.577
	Bicycle lane				
	Off-road	0.941	−0.249	0.499	−1.145
Road width	Less than 8 m (ref.)				
	8–11.9 m	0.815	−0.967	0.322	−1.840
	12–24.9 m	1.149	0.411	0.111	−2.428 *
	25 m or wider	0.881	−0.356	0.121	−2.286 *
Road intersection (1 = Intersection, 0 = Non-intersection)		1.089	0.659	1.030	0.073
Road pavement (1 = Unpaved, 0 = Paved)		8.332	1.910 *		
Land slope	Flat (ref.)				
	Mild slope	1.160	0.727	0.830	−0.410
	Steep slope	0.434	−1.297		
Land use zone	Residential zone (ref.)				
	Commercial zone	1.197	1.195	0.732	−0.842
	Rural/Green zone	1.576	2.559 *	1.626	0.947
N		1132		211	
AIC		2420.355		459.9389	

Note: *, **, *** refer to the statistical significance at the 5%, 1%, and 0.1% level, respectively.

**Table 8:** Location of off-road accidents.

Off-Road	Number of Accidents	Off-Road	Number of Accidents
Other	67 (33.8%)	Square (plaza)	16 (8.1%)
Parking lot	46 (23.2%)	University campus	14 (7.1%)
Apartment complex	32 (16.2%)	Trail	6 (3.0%)
Park	17 (8.6%)	Total	198 (100.0%)

## Data Availability

The data presented in this study are available on request from the corresponding author.
